# Exploring the Intersection of Artificial Intelligence and Metaverse Realities: Implications for Digital Mental Health Services

**DOI:** 10.3390/healthcare14203405

**Published:** 2026-10-12

**Authors:** Antonio Pesqueira

**Affiliations:** Department of Technology, Iscte—Instituto Universitário de Lisboa, 1649-026 Lisbon, Portugal; antonio_pesqueira@iscte-iul.pt

**Keywords:** artificial intelligence, metaverse, mental health support, digital healthcare, Delphi study

## Abstract

**Background/Objectives**: Integrating Artificial Intelligence and Metaverse Realities into Digital Mental Health Services presents novel opportunities for clinical assessment, therapeutic intervention, and continuous rehabilitation. Grounded in dynamic capabilities and sociotechnical systems theories, this study evaluates strategic pathways for integrating Artificial Intelligence and Metaverse Realities into Digital Mental Health Services, analyzes core bioethical and data privacy challenges, and examines the prospective impact of immersive virtual environments on patient engagement and therapeutic outcomes. **Methods**: An iterative three-round Delphi inquiry was conducted with an international panel of 36 multidisciplinary experts spanning clinical psychiatry, Artificial Intelligence and Metaverse Realities software development, digital ethics, and healthcare policy. Standardized, neutral visual scenarios prompted expert evaluations across 14 Digital Mental Health Services sub-areas. Panel consensus, rating dispersion, and domain interrelationships were quantified using Kendall’s coefficient of concordance (W), non-parametric Spearman rank-order correlation analysis, and descriptive dispersion metrics (median, interquartile range, standard deviation), complemented by reflexive thematic qualitative synthesis. **Results**: Expert agreement converged significantly from Round 1 to Round 3. High consensus emerged for AI-enhanced initial screening (mean = 4.7, SD = 0.5) and comprehensive VR assessments (mean = 4.5, SD = 0.8). While AI-managed crisis services showed high perceived probability (95%), findings highlighted the necessity of strict human-in-the-loop architectures over full autonomy to mitigate psychiatric triage hazards. **Conclusions**: Artificial Intelligence and Metaverse Realities present viable adjunctive pathways for personalized psychiatric assessment and therapy, provided that systems implement strict clinician-in-the-loop safeguards, real-time dynamic consent, and end-to-end data encryption. Utilizing a Dynamic Capabilities framework enables healthcare organizations to systematically sense technological innovations, seize clinical opportunities, and reconfigure governance structures, thereby ensuring equitable, safe, and ethical digital mental health delivery.

## 1. Introduction

The integration of Artificial Intelligence (AI) and Metaverse Realities (MR) into Digital Mental Health Services (DMHS) has the potential to offer innovative solutions to longstanding challenges in mental health assessment, diagnosis, intervention, and rehabilitation.

Recently, both AI and MR have emerged as key components in DMHS, with the potential to enhance the assessment and diagnosis phases by analyzing vast datasets. However, this integration poses several challenges in terms of data protection, security, and overall quality. These technologies have great potential to address mental health concerns by offering more precise and personalized diagnoses and by providing patient-supporting services. However, it is essential to ensure that they are effectively aligned with applicable regulations and security protection protocols. Furthermore, both technologies can facilitate the analysis of behavioral data and the simulation of therapeutic scenarios, resulting in customized mental health interventions. In conjunction with other digital technologies, such as telehealth and virtual diagnostics, these technologies can enhance healthcare delivery by reducing waiting times, increasing accessibility, and enabling remote patient monitoring [1,2].

In the context of mental health services, advancements in AI, complemented by Machine Learning (ML) or Natural Language Processing (NLP), hold particular promise. These technologies can enhance assessment and diagnosis by leveraging detailed datasets, including the analysis of large electronic health records (EHRs) and real-time behavioral data. Additionally, MR can create immersive environments that simulate real-life scenarios, enabling clinicians to observe patient behaviors and responses in a controlled setting. This immersive capability provides a richer context for assessment and can enhance the accuracy of diagnoses. In this way, these platforms can significantly improve therapeutic interventions by offering personalized treatment plans and creating engaging, interactive therapeutic environments [3].

Also connected with rehabilitation and recovery, these technologies offer ongoing support, skill-building exercises, and virtual community integration, where AI can track progress and adjust recovery plans, while MR environments facilitate skill development and social interaction [4]. The interoperability of AI subsets, such as ML, NLP, and generative AI (Gen-AI), with MR technologies can create objective frameworks for innovative mental health solutions. However, integrating these technologies must be approached with careful consideration of ethical, legal, and security concerns [5,6]. Ensuring the safety, privacy, and efficacy of users is of the utmost importance. Despite the advancements in AI and digital platforms, current mental health services face challenges such as accessibility, personalization, and inclusiveness [6,7,8,9].

Grounded in Dynamic Capabilities (DC) theory and sociotechnical systems theory, this study investigates how healthcare organizations can systematically sense emerging computational opportunities, seize therapeutic modalities, and reconfigure clinical governance to support DMHS. Despite accelerating investments in AI and MR, three foundational evidence gaps persist: first, an absence of consensus on how multi-modal generative AI and immersive mixed-reality architectures can be integrated into clinical workflows without compromising therapeutic rapport; second, an underdeveloped regulatory understanding of real-time bioethics, dynamic consent, and digital phenotyping privacy; and third, an absence of prioritized implementation roadmaps that balance technical feasibility against acute clinical risk [7,8].

To address these gaps, this study examines three primary research questions: RQ1 (Technological Architecture): Which clinical workflows within DMHS, ranging from diagnostic triage to VR psychotherapy, exhibit the highest feasibility and utility when integrating AI and MR? RQ2 (Bioethical Governance): What systemic frameworks and consent mechanisms are required to safeguard patient autonomy, biometric privacy, and algorithmic safety in immersive therapeutic spaces? RQ3 (Implementation Barriers): What socio-technical and infrastructural disparities impede the equitable delivery of AI-mediated immersive mental healthcare?

To answer these questions, an iterative three-round Delphi inquiry was executed across an international panel of 36 multidisciplinary experts. The study followed an integrated sequential design: Round 1 comprised an exploratory qualitative phase using semi-structured protocols to extract emerging operational domains and bioethical priorities. The thematic outputs of Round 1 directly informed the stimulus engineering of Round 2, wherein panelists evaluated 14 distinct DMHS clinical sub-areas alongside standardized, neutrally prompted visual scenarios to contextualize immersive workflows. Round 3 transitioned to final consensus-building, prioritization ranking, and stability testing.

## 2. Literature Review

### 2.1. Empowerment, Group Cohesion, and Social Impact

Patient empowerment in mental health support is a dynamic process deeply influenced by social interaction and communal support. As individuals develop a greater sense of control over their health decisions and life trajectories, they often rely on meaningful relationships and group settings to further bolster their autonomy and recovery. Research shows that empowerment enhances patients’ agency and sense of self-efficacy, ultimately contributing to improved mental health outcomes [10,11,12]. Still, the translation of empowerment from conceptual ideals into clinical practice can be fraught with complexity, especially when trying to ensure that individuals genuinely participate in care decisions rather than merely following recommendations. Research points to the difficulty of adapting policy-level directives into actionable steps, emphasizing that true empowerment demands not only institutional support but also a willingness to share power with patients. Similarly, the relevance of empowerment in reducing the sense of powerlessness that often exacerbates mental health conditions, thereby supporting the notion that empowerment and social support operate symbiotically to improve mental well-being, is also underlined [13,14,15].

As patients grow more empowered, they naturally seek environments in which they can exercise their evolving autonomy, leading to a collective dimension where group identity and cohesion become equally pivotal. In therapeutic contexts, individuals gravitate toward group-based interventions when they recognize that peers share both their struggles and their aspirations. This process not only validates personal experiences but also reinforces the idea that recovery is collective and interdependent. Researchers have found that when group members feel a strong sense of mutual identification, they experience more comprehensive treatment outcomes [16]. Group cohesion emerges consistently in Cognitive Behavioral Therapy (CBT) principles, whether diagnostic-specific or transdiagnostic, suggesting a universal human need for connection that transcends clinical categories. As a result, the synergy between personal empowerment and shared identity generates a supportive foundation where members encourage each other to remain engaged in therapeutic processes, a phenomenon echoed in recent research observations [17,18,19].

Within these cohesive environments, the principles of social impact theory illustrate how group strength, immediacy, and peer presence shape therapeutic engagement. In digital mental health contexts, the perception of shared experience and mutual validation amplifies motivation to adhere to therapeutic regimens, demonstrating that virtual group spaces can replicate the supportive psychosocial scaffolding traditionally cultivated in face-to-face CBT. Cohesive groups create conditions wherein social influence is amplified, partially explaining why individuals who feel validated by their peers often show heightened motivation to adhere to treatment. These insights align with the premise of those who discuss how a group’s support system can act as a potent catalyst for behavioral change, especially when collective identity is strong. The fundamental theoretical construction of social impact theory is then further explained, with computer simulations demonstrating how shifts in attitudes can occur on a population scale when social factors align favorably. While these findings come from computational models, their application to real-world mental health scenarios suggests the potential for widespread impact if group-based interventions harness cohesive identities and strong social ties [20,21,22,23].

Additionally, the Social Action Theory similarly underscores the interplay between individual choices and communal contexts, positing that personal agency is shaped by social environments that either facilitate or hinder health behavior change. Thus, combining empowerment, group cohesion, and social impact principles can guide mental health practitioners in creating interventions that resonate both personally and collectively. When individuals feel empowered, join supportive groups, and experience the potent influence of cohesive networks, they become more open to sustained change. In practice, this means that mental health programs can strategically harness these factors by designing group activities that reinforce shared goals, strengthen peer relationships, and encourage constructive accountability [24,25,26].

By embedding social impact considerations into mental health interventions, practitioners can amplify the efficacy of group support. This might involve structuring therapy groups to ensure consistent attendance, fostering strong bonds among members, and using collaborative goal-setting to strengthen group identity. For example, interventions that incorporate peer mentoring or participant-led discussions often leverage the direct influence peers have on one another, thereby merging the concept of individual empowerment with collective transformation. Recent research suggests that this strategic intertwining of personal and group elements is key for long-lasting outcomes, as it not only addresses the psychological needs of individuals but also integrates communal factors that reinforce positive behavior in broader social settings [27,28,29,30].

The importance of these insights extends to modern healthcare delivery, especially as DMHS gains prominence. Online platforms and virtual communities can replicate, and in some cases augment, the advantages of group cohesion and social influence, though questions remain about how best to preserve authenticity and depth in virtual interactions. The digital context may allow participants who are geographically isolated or socially anxious to join supportive networks more easily, thus broadening access to empowerment-driven interventions [31,32,33,34,35]. Virtual tools must be carefully designed to facilitate genuine connection, addressing the nuanced differences between in-person and online engagement to maintain high levels of group identification and influence. Overall, the convergence of empowerment, group cohesion, and social impact theory in mental health support underscores the value of social factors in driving meaningful behavior change. Each of these elements connects fluidly to the others, creating a cohesive framework for both understanding and improving mental health outcomes among individuals facing depression, anxiety, or related challenges. The existing body of literature points to a clear takeaway: interventions that reinforce agency at the individual level, encourage strong bonds within groups, and tap into the power of social influence are more likely to produce lasting benefits. Researchers and practitioners alike continue to explore these relationships across diverse settings, aiming to refine the models and techniques that best harness social dynamics for therapeutic gain. In doing so, the mental health field moves closer to interventions that not only treat symptoms but also empower individuals to sustain well-being through supportive communities [36,37].

### 2.2. Confluence of Generative AI, the Metaverse, and Mental Health Support

The integration of AI in mental health practices marks a profound shift toward more personalized and precise care. By employing advanced algorithms to tailor interventions to individual needs, AI-driven solutions hold promise for enhancing treatment accuracy and efficacy. Also, rapid technological advancements, particularly the emergence of Gen-AI, introduce complex challenges related to data management and patient confidentiality. In mental health contexts, how sensitive information is collected, stored, and applied gains heightened importance, fueling ethical debates around privacy and user autonomy in an age of expansive digital footprints [13,18,19,20].

Recent developments point to end-to-end encryption, secure data storage protocols, and decentralized frameworks (e.g., blockchain) as potential safeguards within AI-driven Metaverse applications. These approaches bolster transparency and user control over data, ensuring that mental health interventions remain confidential. Yet, the dynamic and immersive nature of MR, where individuals engage through avatars in shared virtual spaces, and AI-mediated interactions necessitate adaptive privacy mechanisms, such as dynamic consent models. These models allow participants to adjust data-sharing preferences in real time, ensuring that privacy settings evolve in tandem with changing therapeutic or social contexts [38,39,40].

The immersive environments offered by MR have shown promise for mental health assessments and interventions. By simulating real-world conditions or crafting specialized therapeutic settings, MR tools can enable clinicians to collect detailed observations on client behavior, provide customized interventions, and monitor progress over time. Within these digitally mediated spaces, patient autonomy and informed consent become paramount, particularly in the realm of DMHS. DMHS encompasses teletherapy, AI-guided therapy, and virtual reality (VR)-based interventions, all of which aim to overcome traditional barriers, such as geography and stigma, by offering accessible, tailored mental health care on digital platforms [41,42,43,44,45]. As AI and MR converge, ethical principles surrounding privacy, inclusivity, and autonomy come to the forefront. Striking a balance between leveraging these new technological capabilities and upholding patient rights remains a central concern. Scholars emphasize that as digital mental health tools evolve, they must adhere to ethical standards that protect vulnerable populations and honor established norms in clinical practice [43,44,45]. This imperative necessitates interdisciplinary collaborations among technologists, clinicians, ethicists, and policymakers, thereby ensuring that innovation proceeds with caution and that patient well-being is at the core of development [46,47,48,49,50].

The integration of Gen-AI within MR environments has unlocked the potential for dynamic, responsive spaces that adjust to each individual’s mental health needs. Early studies have documented the capacity of AI-driven platforms to provide personalized therapeutic content for concerns such as depression and anxiety. While some research suggests these interventions can reduce symptom severity and foster a sense of social connection, the variability in outcomes underscores the need for further inquiry. Factors such as user engagement, cultural relevance, and technological literacy can all influence the effectiveness of AI- and MR-based treatments [48,49,50,51].

Patient engagement and accessibility also remain pivotal considerations. Though MR’s immersive interfaces can boost user involvement, potentially increasing treatment adherence, equity issues persist, including the digital divide and disparate internet access. Scholars consequently highlight the importance of designing interventions that accommodate diverse populations and technical competencies [29]. Regulatory frameworks must likewise protect patient rights, with discussions often focusing on user consent, data security, and the potential for exploitative scenarios if ethical guidelines are not rigorously followed [50].

Over the years, research on digital mental health interventions has moved from initial explorations of teletherapy and web-based counseling to more sophisticated applications involving AI, VR, and immersive Metaverse platforms [45,46,47,48,49,50,51]. These innovations include AI-driven diagnostic tools, VR therapy sessions, and emerging best practices for ethical oversight [43]. As scholars map the current research landscape, they highlight the importance of multi-disciplinary approaches that unite technology, psychology, data ethics, and clinical experience [31,38]. This amalgamation is deemed essential for advancing evidence-based treatments while addressing the inherent complexities of digital environments. Within these developments, DMHS has increased the relevance of assessment, diagnosis, treatment planning, and rehabilitation [49,50,51,52]. By expanding care options and providing continuous monitoring, digital platforms offer early interventions for a range of mental health conditions [36]. Studies have shown that digital assessments, whether through online questionnaires or VR simulations, can be as reliable as traditional face-to-face evaluations. Tailored treatment plans, powered by AI analytics, adapt to individual symptoms and evolve with patient progress. Systematic reviews and meta-analyses generally confirm the efficacy of digital therapies in managing common mental health disorders, while underscoring the need for data security practices [19,39,40,41,42].

As digital mental health interventions proliferate, they hold particular promise for chronic conditions requiring ongoing support. The literature indicates that therapeutic continuity and regular progress tracking can bolster outcomes in areas like depression, anxiety, and post-traumatic stress disorder. Yet user engagement, adherence, and long-term cost-effectiveness are not always guaranteed. Studies examining interface design, motivational strategies, and economic factors highlight critical gaps that must be addressed to facilitate broad-based adoption. Looking back at how DMHS emerged from early telemedicine to contemporary AI- and VR-integrated models reveals a consistent trend: the quest for greater accessibility, personalization, and scalability in mental health care. The potential for these services to democratize mental health support is evident, but significant effort remains to ensure equitable distribution, evidence of efficacy, and stringent ethical safeguards. The trajectory of these innovations suggests that future research should emphasize cost-effectiveness analyses, user experience design, and the creation of inclusive frameworks that protect patient rights across diverse contexts. Thus, the evolving confluence of AI, Gen-AI, and MR signals a transformative era in mental health support. From personalized VR treatment sessions to AI-enhanced diagnostic tools, digital platforms are redefining how we understand, treat, and monitor mental health conditions. Nevertheless, ethical and practical considerations, especially around privacy, informed consent, and equitable access, must guide these technological endeavors. As the field continues to expand, a multidisciplinary focus and a commitment to ethical standards will be essential to fully harness the benefits of these digital innovations without compromising the core principles of patient-centric care [51,52].

## 3. Methodological Approach

### 3.1. Study Design and Delphi Architecture

A sequential three-round Delphi design was deployed to synthesize consensus across disparate disciplines, circumvent peer-conformity bias, and eliminate interpersonal hierarchies inherent in unstructured focus groups. To maintain procedural clarity and methodological consistency, the research process is structured across three sequential Delphi rounds: Round 1 (Exploratory Qualitative Scoping): Identification of key operational domains, clinical challenges, and bioethical priorities via semi-structured expert inquiries. Round 2 (Standardized Stimulus Evaluation & Likert Rating): Presentation of 14 specific clinical sub-areas paired with standardized, neutral-valence AI-rendered clinical scenarios to evaluate feasibility, probability, and therapeutic impact. Round 3 (Consensus Convergence & Prioritization): Iterative re-evaluation of items based on aggregate panel feedback, statistical stability testing, and priority ranking.

### 3.2. Delphi Study Conceptualization and Questionnaire Design

Candidate recruitment followed purposive sampling criteria to balance technical expertise, clinical psychiatry, bioethics, and healthcare governance. Expert inclusion required meeting at least two of the following criteria: (a) a minimum of five years of active professional practice in clinical psychiatry, psychological counseling, medical informatics, or health systems engineering; (b) peer-reviewed publications addressing digital health, machine learning in medicine, or immersive VR therapy; or (c) active leadership roles in digital health policymaking or bioethics committees. The recruitment trajectory progressed through a transparent four-stage pipeline:

1. **Initial Identification:** Ninety-seven (*n* = 97) prospective international candidates were identified via institutional registries, academic citation indexing, and professional medical technology societies.
2. **Formal Invitation and Screening:** Personalized formal invitations and eligibility screening surveys were dispatched; 23 candidates declined due to scheduling constraints or declared conflicts of interest, and 18 failed to respond, leaving 56 screened candidates.
3. **Pre-Delphi Onboarding:** Of the 56 eligible candidates, 41 confirmed participation and completed dynamic informed consent declarations. Five withdrew before Round 1 due to professional workload reallocations.
4. **Final Analytic Sample:** A panel of *n* = 36 experts fully completed Round 1. To preserve the statistical validity of the iterative Delphi process, strict panel retention protocols were maintained, achieving a 100% longitudinal completion rate across Round 2 (*n* = 36) and Round 3 (*n* = 36).

The panel comprised four disciplinary domains: clinical psychiatrists and psychotherapists (*n* = 14, 38.9%), AI and XR healthcare software engineers (*n* = 11, 30.6%), digital bioethicists and health legal scholars (*n* = 7, 19.4%), and digital health policymakers/administrators (*n* = 4, 11.1%). In the exploratory qualitative phase (Round 1), sampling adequacy was governed by thematic saturation principles, with saturation confirmed by the 28th interview transcript, after which no novel conceptual codes were identified across the remaining eight transcripts [7,8,9,42].

### 3.3. Round 1: Qualitative Protocol and Thematic Coding

Round 1 gathered qualitative insights regarding the perceived clinical efficacy, systemic risks, and governance requirements of AI and MR integration in DMHS. Data collection was administered via a secure, web-based digital platform hosting an open-ended, semi-structured qualitative questionnaire (the complete protocol is provided in Supplementary File S1 and Appendix A). The instrument was developed following a complete literature review of sociotechnical systems in healthcare and piloted with two independent academic psychiatrists to establish face and content validity.

The qualitative inquiry guide was derived from foundational literature spanning sociotechnical transitions in clinical psychiatry and dynamic digital governance. The interview protocol systematically operationalized four dimensions: (1) Emergent therapeutic affordances in MR environments, informed by Riva et al.’s cyberpsychology scaffolding and Freeman et al.’s virtual reality psychotherapy paradigms [32,33]; (2) Clinical risks and algorithmic autonomy in diagnostic triage, structured around Sedlakova and Trachsel’s bioethical boundaries for conversational agents and computerized triage [48]; (3) Biometric data privacy, digital phenotyping, and dynamic consent, adapted from emerging neuroethics and precision digital health privacy frameworks [29,37,41]; and (4) Structural disparities, digital divides, and equitable access barriers, formulated via sociotechnical health equity constructs [7,40]. The resulting semi-structured questions explored organizational sensing, seizing, and governance reconfiguration capabilities across each domain. Participants completed the protocol asynchronously, with an average active completion time of 38 min (SD = 9.4). All qualitative sessions were managed and curated by the lead researcher, who holds a doctorate in health information systems and professional training in qualitative health methodology.

Transcribed qualitative responses were analyzed following the six-phase reflexive thematic analysis framework articulated by Braun and Clarke [52]. The analytical progression adhered strictly to the established stages: (Phase 1) data familiarization through repetitive reading and active transcript annotation; (Phase 2) generating initial codes across semantic and latent expert statements; (Phase 3) searching for candidate themes by clustering related conceptual codes; (Phase 4) reviewing and refining candidate themes against both individual extracted coded extracts and the global data corpus; (Phase 5) defining and naming themes to ensure clear operational conceptualization; and (Phase 6) producing the final analytical narrative contextualized with representative data exemplars. NVivo 14 qualitative data analysis software (Lumivero, Denver, CO, USA) was utilized as the computational platform to manage data organization and enhance analytical auditability. Specifically, NVivo was employed to: (a) structure an a priori deductive coding hierarchy based on Dynamic Capabilities Theory (parent nodes: Sensing, Seizing, and Reconfiguring); (b) map inductively surfaced open codes (child nodes) derived directly from participant statements; (c) execute text search and word frequency queries to cross-validate emerging patterns; and (d) generate coding comparison queries between two independent coders across a randomized 30% sample of raw transcripts to calculate Cohen’s kappa (*κ* = 0.84). Conceptual discrepancies were addressed through iterative reflexive consensus sessions until 100% agreement on node definitions was achieved. The finalized thematic codebook directly informed the 14 DMHS sub-areas evaluated in Round 2 [7,8,9,53].

### 3.4. Round 2: Standardized Visual Scenario Construction and Stimulus Neutrality

To provide standardized clinical contexts for expert rating, Round 2 incorporated six visual scenarios depicting AI and MR workflows (e.g., automated psychiatric intake triage, immersive exposure therapy, multidisciplinary virtual case conferences). Following recent methodological frameworks for systematic AI prompt engineering and output evaluation in psychological contexts, visual stimuli were constructed through a controlled, reproducible pipeline rather than ad hoc generation (provided in Supplementary File S2).

Image prompts were generated using Midjourney v5.2 (Midjourney Inc., San Francisco, CA, USA). Prompt syntax followed a uniform grammatical formula: ‘[Clinical Setting/Context] + [Actor/Patient Interaction] + [Technological Modality] + [Stylistic and Architectural Parameters] + [Engine Parameters]’. To prevent affective framing effects, prompts were restricted to emotionally neutral descriptors, balanced lighting, and desaturated, non-clinical color temperatures (e.g., “minimalist, neutral daylight illumination, photorealistic, matte architectural finish”). Midjourney rendering parameters were held strictly constant across all generations to ensure aesthetic standardization: Aspect Ratio (‘--ar 16:9’), Stylize (‘--s 50’, representing low algorithmic interpretation), Chaos (‘--c 0’, minimizing randomness), and Version (‘--v 5.2’).

Stimulus neutrality was validated through a pre-evaluation protocol conducted by an independent review panel of three senior academic clinicians (two psychiatrists, one clinical psychologist) not involved in the subsequent Delphi panel. The reviewers independently rated each rendered image across three 5-point semantic differential scales adapted from visual psychophysics: Affective Valence (1 = highly negative/distressing, 3 = strictly neutral, 5 = highly positive/euphoric), Visual Complexity (1 = sparse/understimulating, 3 = balanced, 5 = cluttered/overstimulating), and Clinical Realism (1 = implausible fiction, 5 = highly representative of near-term clinical settings). Kendall’s coefficient of concordance among the three reviewers demonstrated high pre-evaluation agreement across the image suite (W = 0.88, *p* < 0.01). Images deviating from the target neutrality window (affective valence score outside 3.0 ± 0.5) underwent single-parameter prompt revision and iterative regeneration until consensus on visual neutrality was established.

### 3.5. Round 3: Statistical Metrics and Consensus Benchmarks

Quantitative responses from Rounds 2 and 3 were analyzed using SPSS Statistics Version 29.0 (IBM Corp., Armonk, NY, USA) and R Version 4.3.2 (R Foundation for Statistical Computing, Vienna, Austria). Descriptive central tendencies and dispersion metrics were calculated for each of the 14 DMHS sub-areas: median, mean, standard deviation (SD), and interquartile range (IQR). Predefined criteria established consensus stability across rounds: an interquartile range (IQR) ≤ 1.0 accompanied by a standard deviation (SD) < 0.80 denoted high item consensus. Panel-wide concordance across the 14 sub-areas was assessed using Kendall’s coefficient of concordance ($W=\frac{12S}{k^{2}\left(n^{3}-n\right)}$) where S represents the sum of squared deviations of item rank totals from the mean rank total, k is the number of expert raters (*k* = 36), and n is the total number of evaluated sub-areas (*n* = 14). Statistical significance was evaluated using the chi-square approximation with 13 degrees of freedom (df = *n* − 1 = 13). Because Delphi ratings derive from 5-point ordinal Likert scales that violate multivariate normality assumptions, bivariate interrelationships among DMHS domains were quantified using non-parametric Spearman rank-order correlation coefficients (ρ) rather than parametric Pearson metrics. Statistical significance was set at α = 0.05, accompanied by two-tailed 95% confidence intervals.

## 4. Results

### 4.1. Round 1 Qualitative Findings

The inductive–deductive thematic coding of Round 1 transcripts identified eight prominent themes characterizing the integration of AI and MR into DMHS. Table 1 outlines these thematic domains, associated operational descriptions, and representative expert exemplars. Panelists consistently emphasized that technological viability in automated screening and immersive exposure must be balanced against systemic concerns regarding algorithmic bias, non-transparent diagnostic recommendations, and the dilution of therapeutic presence.

Synthesizing expertise across computer science, clinical psychology, and regulatory bioethics is essential to devise governance models that protect user autonomy and data privacy without stifling technological innovation. This collaborative approach has the potential to facilitate the development of practical, ethical, and effective solutions. Key areas that warrant attention include the ethical considerations surrounding privacy, confidentiality, and user autonomy concerns associated with the integration of advanced technologies in mental health services. In addressing the challenges associated with the implementation of AI and ML in mental health services, it is imperative to recognize the multifaceted nature of these challenges. These challenges encompass technical, infrastructural, and user acceptance aspects, necessitating a collaborative approach that engages a diverse array of stakeholders. Continuous research and dialogue are essential to ensure the effective integration of these technologies into various clinical and therapeutic contexts. In doing so, future projections and assumptions must be considered, with a focus on the creation of comprehensive ethical frameworks and the equitable distribution of benefits (as Table 1).

### 4.2. Descriptive Statistics

**Figure 1:**
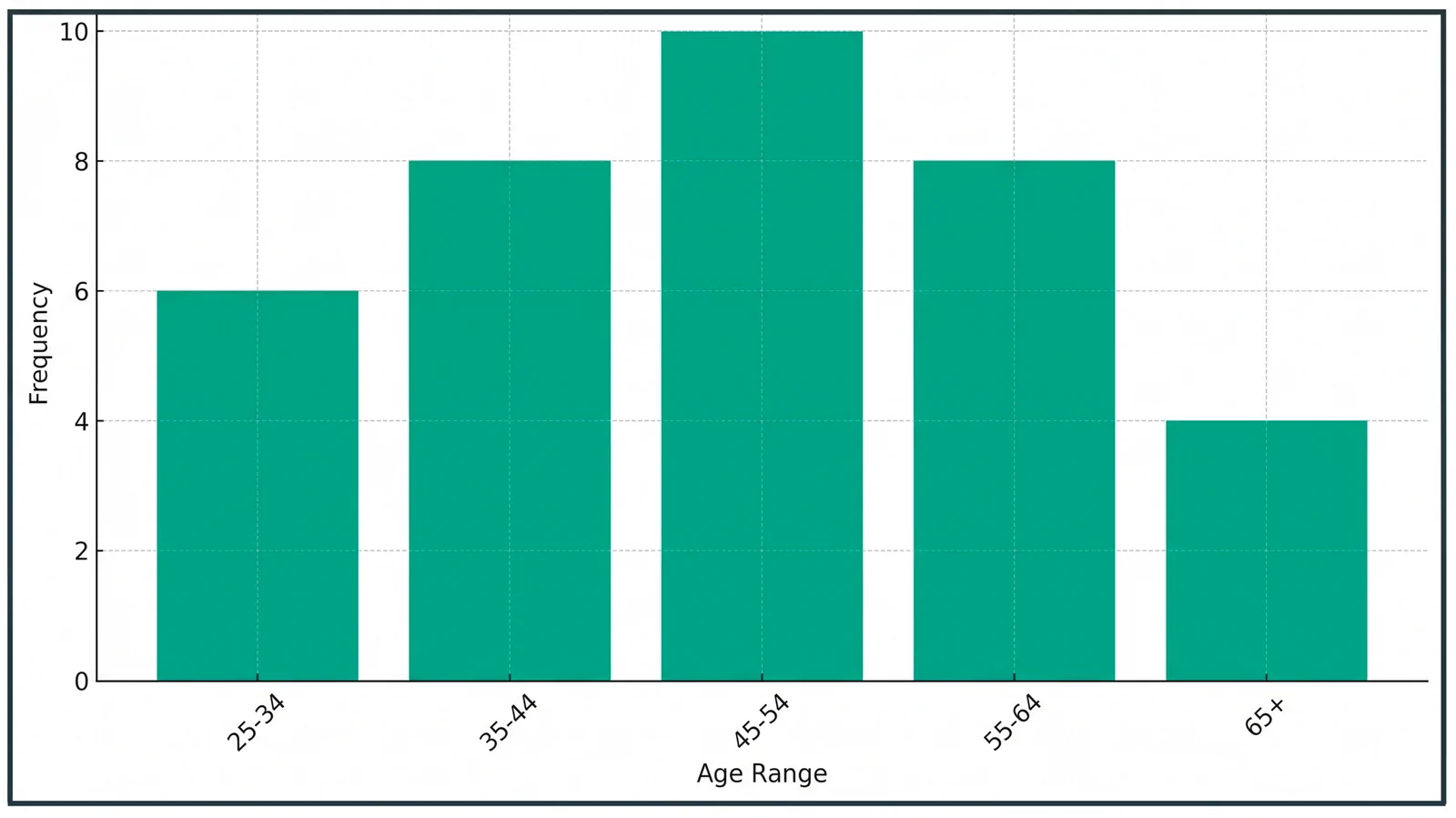
Age distribution of healthcare mental health professionals.

The age distribution of the 36 participants demonstrates balanced professional representation across early-, mid-, and senior-career stages (see Figure 1). Categorized into five distinct ranges (25–34, 35–44, 45–54, 55–64, and 65+), the data indicate that mid-career professionals aged 45–54 represent the largest cohort (*n* = 10, 27.8%), followed by cohorts aged 35–44 (*n* = 8, 22.2%) and 55–64 (*n* = 8, 22.2%), ensuring both clinical experience and technological openness across the sample.

**Figure 2:**
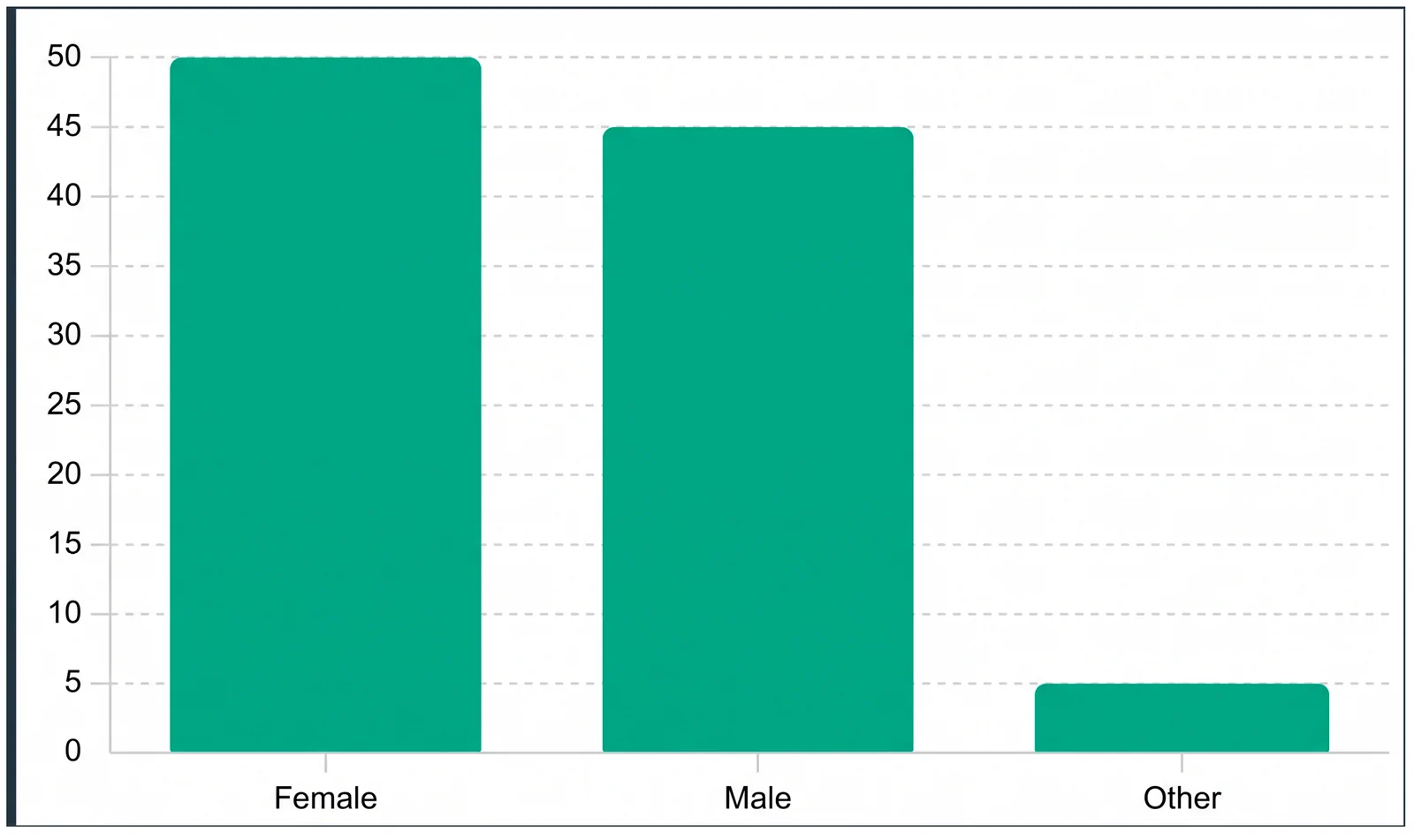
Gender distribution of healthcare mental health professionals.

The gender distribution chart depicts the representation of different genders among the healthcare mental health professionals sampled (Figure 2). Females constitute the largest segment at 50%, followed closely by males at 45%. This nearly equal representation ensures that the study captures insights from both male and female perspectives, which is crucial for balanced gender considerations in healthcare. Additionally, 5% of the sample identifies as ‘Other,’ highlighting the inclusion of non-binary or gender-diverse individuals. This representation is significant as it acknowledges and incorporates the perspectives of professionals who do not identify strictly as male or female, promoting inclusivity and diversity within the study.

**Figure 3:**
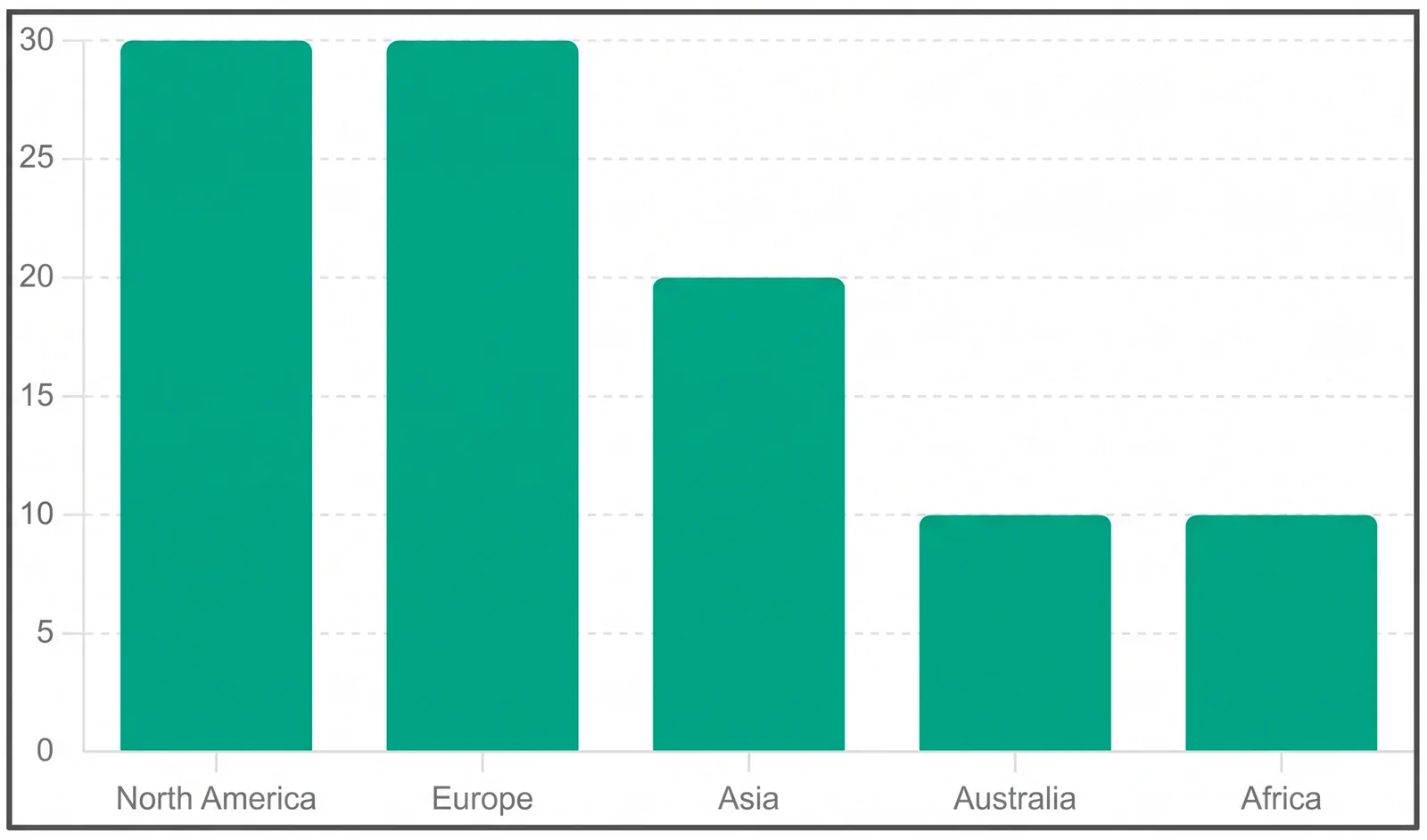
Location distribution of healthcare mental health professionals.

The location distribution chart illustrates the geographical diversity of the sample (see Figure 3). North America and Europe each represent 30% of the sample, indicating a strong presence of professionals from these regions. Asia follows with 20%, contributing a substantial portion of the sample. Australia and Africa each represent 10%, showing participation from these continents as well. This geographical diversity is essential for capturing a wide range of cultural and healthcare contexts, which can enrich the study’s findings and ensure that the insights and conclusions are relevant across different regions. The varied locations of participants underscore the global nature of the study and its applicability to diverse healthcare settings.

### 4.3. Future Projections

The following table (see Table 2) presents an in-depth examination of the projected impact and likelihood of various AI and Metaverse applications in DMHS, assessed using a Likert scale. The table enumerates several projections, each identified by an ID number, describing distinct applications and their respective impact and probability scores. AI-enhanced initial Screening aims to analyze initial screening data to identify subtle mental health indicators, with an impact score of 4.1 and a 74% probability, using voice patterns and behavioral data from Metaverse interactions.

Comprehensive Assessment via VR enables thorough evaluations in immersive VR environments, scoring 3.9 in impact and 81% in probability. AI-Assisted Diagnosis integrates comprehensive assessments and medical history to provide diagnostic recommendations, scoring 4.7 in impact and 86% in probability. Dynamic Individualized Treatment Plans, updated in real-time from Metaverse interactions, present a significant advancement with an impact score of 4.6 and a 79% probability. Enhanced Multidisciplinary Collaboration through virtual workspaces and AI-coordinated care plans fosters seamless healthcare team cooperation, with an impact score of 4.7 and a 75% probability. AI-optimized pharmacotherapy adjusts medication dosages in real-time, scoring 4.1 in impact and 84% in probability. VR Psychotherapy delivers immersive therapeutic environments, including exposure therapy, scoring 4.7 in impact and 90% in probability. AI-guided counseling and Support offers continuous support within the Metaverse, with an impact score of 4.1 and a 64% probability. Virtual Emergency Services provide immediate virtual crisis intervention, scoring 4.3 in impact and 87% in probability. AI-Managed Crisis Hotlines, with a 4.9 impact score and 95% probability, use chatbots for managing crisis support. Virtual Rehabilitation Programs, offering vocational training and social skills development, score 3.7 in impact and 57% in probability. AI-Facilitated Peer Support Networks create virtual support groups, scoring 4.3 in impact and 78% in probability. Automated Relapse Prevention Strategies monitor activities to prevent relapse, scoring 3.7 in impact and 60% in probability. Longitudinal Impact Studies stress the need to understand long-term effects, scoring 3.6 in impact and 74% in probability.

Whereas Table 2 details the scenario-specific impact and perceived occurrence probability elicited during Round 2, longitudinal consensus convergence across all three Delphi rounds was evaluated across seven core thematic areas, as reported in Table 3 (displaying Interquartile Range [IQR], Mean, and Standard Deviation [SD] per round). The primary domains comprise Assessment and Diagnosis, Treatment Planning and Coordination, Intervention and Therapy, Crisis Intervention, Rehabilitation and Recovery, Ethical and Privacy Concerns, and Accessibility and Inclusivity. In the key areas of Assessment and Diagnosis, “AI-Enhanced Initial Screening” shows a high level of consensus and importance, with the mean increasing from 4.4 in Round 1 to 4.7 in Round 3, and the IQR remaining low. “Comprehensive Assessment via VR” also demonstrates increasing consensus and importance, with the mean rising from 3.9 to 4.5 across the rounds. In Treatment Planning and Coordination, “Dynamic Individualized Treatment Plans” maintain a high mean score (around 4.1), indicating its significance, with consistent agreement (IQR of 1 across rounds). “Enhanced Multidisciplinary Collaboration” shows strong consensus and importance, with the mean increasing slightly to 4.3 in Round 3. In Intervention and Therapy, “Virtual Reality Psychotherapy” has a mean that rises to 3.8 by Round 3, reflecting its growing perceived importance. “AI-Guided Counseling and Support” also shows an increased consensus, with the mean reaching 4.0 in the final round. In Crisis Intervention, “Virtual Emergency Services” has a lower mean score of 3.5 by Round 3, indicating moderate importance. “AI-Managed Crisis Hotlines and Support Lines” shows a high mean score of 4.2 in the final round, reflecting a strong consensus on its importance. In Rehabilitation and Recovery, “Virtual Rehabilitation Programs” maintain a steady mean score of around 4.0, indicating high importance and consensus. “AI-Facilitated Peer Support Networks” shows a strong consensus, with the mean reaching 4.3 by Round 3. In Ethical and Privacy Concerns, “Ensuring User Safety and Privacy” has a high mean score of 4.4 in the final round, reflecting the critical importance of this sub-area. “Addressing Ethical and Legal Concerns” shows increasing importance, with the mean reaching 4.3 in Round 3. In Accessibility and Inclusivity, “Bridging the Digital Divide” shows a consistent mean score of around 4.0, indicating its significant relevance. “Ensuring Cultural Sensitivity” maintains a high mean score of 4.1, reflecting a strong consensus on its importance. The statistical measures across three rounds demonstrate increasing agreement and highlight the critical areas for future research and implementation.

### 4.4. Consensus Convergence and Domain Correlation Analysis

Omnibus concordance among the 36 experts increased substantially across successive rounds, progressing from moderate agreement in Round 1 (W = 0.46, χ^2^(13) = 215.28, *p* < 0.001) to strong concordance by Round 3 (W = 0.74, χ^2^(13) = 346.32, *p* < 0.001, df = 13), confirming panel convergence on clinical priorities.

**Figure 4:**
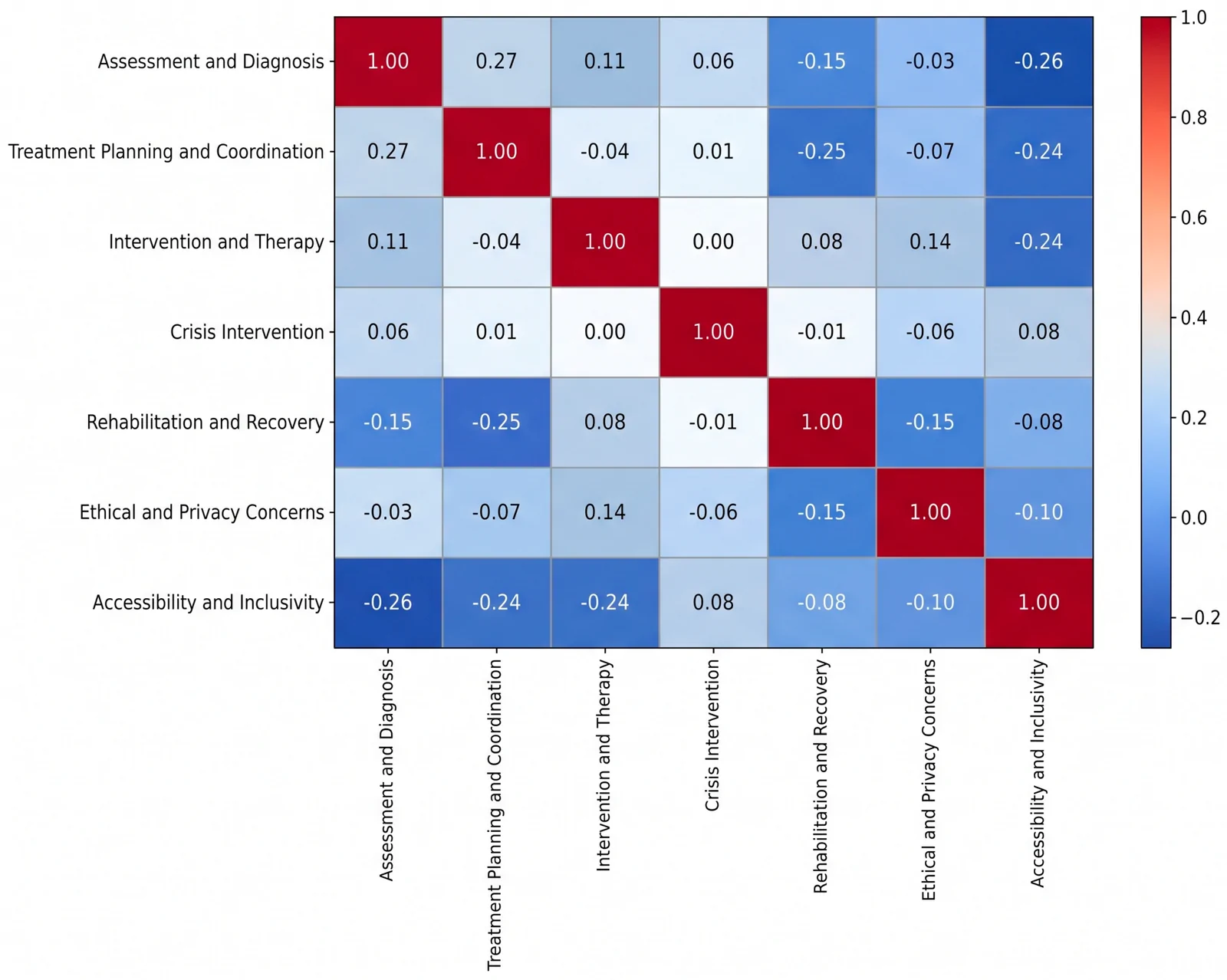
Domain Correlation Matrix. Values within the matrix display aggregate bivariate correlation coefficients across grouped clinical domains; the accompanying text reports confirmed non-parametric Spearman rank correlation coefficients (*ρ*) with 95% confidence intervals.

Figure 4 presents the aggregate bivariate Spearman rank correlation matrix (ρ) across the primary DMHS domains. The non-parametric analysis reveals distinct operational independence among several domains. Support for Assessment and Diagnosis correlates weakly with Intervention and Therapy (ρ = 0.12, 95% CI [−0.22, 0.43], *p* = 0.485), indicating that expert prioritization of automated intake screening operates independently from preferences for virtual psychotherapeutic interventions. Treatment Planning and Coordination demonstrates a negligible association with Ethical and Privacy Concerns (ρ = −0.07, 95% CI [−0.38, 0.27], *p* = 0.728). Notably, Accessibility and Inclusivity exhibits an inverse relationship with both Assessment and Diagnosis (ρ = −0.26, 95% CI [−0.56, 0.05], *p* = 0.098) and Intervention and Therapy (ρ = −0.24, 95% CI [−0.53, 0.08], *p* = 0.11). Although these rank correlations do not achieve statistical significance at the two-tailed α = 0.05 threshold, the negative directionality reflects panel apprehension that high-bandwidth, hardware-intensive metaverse deployments risk widening disparities in care access for resource-constrained populations.

## 5. Discussion

### 5.1. Translating Consensus into Clinical Reality: The DC Lens

The Delphi panel reached high consensus regarding the prospective utility of AI and MR across specific DMHS applications, particularly automated intake screening (mean = 4.7, SD = 0.5) and immersive VR exposure assessments (mean = 4.5, SD = 0.8). However, these findings capture structured expert projections rather than validated clinical efficacy. Expert alignment regarding technological plausibility must not be conflated with empirical therapeutic equivalence to gold-standard psychiatric care. Translating these emergent capabilities into real-world health services requires navigating the systemic tensions identified by the panel. A salient example is the expert projection regarding AI-managed crisis hotlines, which garnered an impact score of 4.9 and a perceived probability of 95%. While this highlights a perceived urgency to mitigate capacity shortages in acute crisis services, deploying autonomous conversational agents in psychiatric triage introduces severe clinical hazards. Natural language processing models remain susceptible to semantic hallucination, algorithmic confabulation, and affective misattunement during acute suicidal ideation. Rather than full autonomy, these consensus findings must be operationalized through strict “human-in-the-loop” triage architectures, wherein AI systems serve exclusively as diagnostic copilots that extract linguistic markers of risk to accelerate referral to licensed clinicians. This approach addresses the core priorities emphasized by the panel: expanding therapeutic accessibility while enforcing clinical safeguards to protect patient privacy, data integrity, and therapeutic rapport. Here, the DC framework provides actionable guidance. Rather than implementing AI and MR as static digital infrastructure, healthcare organizations must build tripartite agility: 1. Sensing: Establishing continuous surveillance mechanisms to track algorithmic drift, biometric data validity, and clinical safety signals across emerging psychiatric software-as-a-medical-device (SaMD) platforms. 2. Seizing: Structuring validated clinical protocols and interdisciplinary care pathways where clinicians co-deliver VR interventions with calibrated AI assistance. 3. Reconfiguring: Redesigning institutional governance to support dynamic consent architectures, decentralized health records, and continuous professional retraining in algorithmic literacy.

These findings demonstrate that realizing clinical utility in immersive DMHS depends entirely on embedding operational safeguards, specifically continuous clinical oversight and dynamic consent, directly into the underlying software architecture. However, clinical efficacy and technological maturity cannot be evaluated in isolation from structural health systems constraints. Grounding these architectural safeguards within the dynamic capabilities lens highlights that organizational reconfiguration must extend beyond internal software routines to address socio-structural barriers. Panelists repeatedly emphasized that deploying advanced AI and MR architectures within underfunded or unevenly equipped healthcare settings risks exacerbating the existing digital divide. When therapeutic immersion requires costly head-mounted displays and high-speed broadband, vulnerable patient cohorts face systemic disenfranchisement. Consequently, healthcare organizations must cultivate seizing and reconfiguring capabilities that unite multi-sector stakeholders, clinicians, engineers, and healthcare policymakers, to subsidize necessary hardware, deliver culturally adapted virtual environments, and formulate inclusive access policies. In tandem with infrastructural access, immersive virtual interactions introduce unprecedented governance challenges surrounding biometric privacy and digital phenotyping. Because metaverse platforms continuously capture granular behavioral telemetry, such as pupillary dilation, voice inflections, and kinetic responses, static, one-time consent agreements are fundamentally inadequate. Panel consensus underscored that healthcare institutions must reconfigure clinical governance around real-time, dynamic consent mechanisms. These protocols empower patients to grant, withhold, or revoke access to specific behavioral data streams continuously as their therapeutic needs evolve. Finally, overcoming steep clinician learning curves and hardware ergonomic limitations demands structured digital literacy initiatives. By intertwining algorithmic safety, infrastructural equity, and responsive bioethical governance through an overarching dynamic capabilities framework, health systems can responsibly deploy AI-mediated immersive mental health services without compromising patient welfare or equitable care.

### 5.2. Practical Applications of AI and Metaverse Technologies in Mental Health

The integration of AI and MR in mental health services offers a compelling opportunity to reshape the delivery of care, expand personalization, and broaden accessibility. By combining the analytic strengths of AI with the immersive qualities of MR environments, new dimensions of mental health support emerge that go beyond traditional clinical settings. Early screening tools, for instance, can deploy advanced language processing techniques to identify subtle shifts in speech patterns, sentiment, and emotional cues, enabling more precise, real-time assessments of conditions such as anxiety and depression. When paired with MR platforms, these assessments become even more contextualized, as patient interactions within virtual environments can yield nuanced data about behavioral responses that may be overlooked in standard questionnaires or face-to-face evaluations.

Beyond initial screening, VR scenarios provide unprecedented possibilities for in-depth clinical assessments. Clinicians can introduce carefully controlled simulations that emulate fear-inducing or stressful conditions, allowing them to observe patient reactions without placing anyone in actual danger. Individuals struggling with specific phobias or social anxiety can engage with immersive environments tailored to their unique challenges, thereby generating more accurate data on their emotional and physiological states. AI-driven diagnostic tools integrate and interpret diverse data sources, including electronic health records, real-time behavioral inputs, and self-report measures, to produce comprehensive clinical insights. These insights can guide providers in developing individualized treatment pathways that evolve dynamically as patients progress, ensuring that therapeutic goals remain both actionable and responsive to changing needs. AI’s potential to adapt interventions over time significantly enhances therapeutic efficacy. Machine learning algorithms can continuously monitor user engagement, detect subtle shifts in mood, and refine treatment strategies to maintain efficacy. By combining these adaptive capabilities with MR-based settings, mental health professionals can deliver interventions where the virtual environment is adjusted in response to patient feedback. Individuals undergoing exposure therapy for trauma, for example, might experience graduated levels of virtual stimulation, with the system modulating intensity in real time. This personalized fine-tuning mitigates unnecessary distress and fosters sustained engagement, a critical factor in successful mental health outcomes. Moreover, the social dimensions of MR create avenues for group-based interventions and peer support networks. Virtual community spaces, facilitated by AI moderation tools, enable safer environments for individuals to share experiences and overcome isolation. These networks can also integrate real-time crisis support, where AI-powered screening systems detect signs of acute distress and swiftly connect users to professional help. Interdisciplinary research underscores the importance of such immediate responsiveness, particularly when dealing with at-risk populations unwilling or unable to access traditional care settings. Also relevant is the use of immersive platforms for rehabilitation and skill-building exercises, such as practicing job interviews or public speaking in carefully calibrated virtual scenarios that mirror real-world settings while preserving psychological safety. Ongoing data collection within MR environments promises deeper insights into longitudinal outcomes, enabling researchers and clinicians to refine their methods through iterative feedback loops. The capacity to capture and analyze patterns of user interaction across time holds significant promise for both personalized care and large-scale epidemiological studies. Nonetheless, the successful implementation of AI and MR also depends on ethical governance that addresses privacy, data security, and equitable access. In regions lacking digital infrastructure, for instance, developers and policymakers must collaborate to minimize barriers, thus ensuring that these emergent technologies do not widen existing gaps in mental health provision. Adopting a flexible, future-oriented mindset, one that senses, seizes, and transforms in response to technological progress, can help organizations and practitioners leverage AI and MR responsibly. By aligning these innovations with principles of user autonomy, informed consent, and inclusivity, the field of mental health care can evolve toward more responsive and impactful interventions that match the complexity of patients’ lived experiences.

## 6. Limitations and Future Directions

### 6.1. Study Limitations

The findings of this investigation must be interpreted in light of several methodological limitations:

1. Perceptual vs. Empirical Evidence: The data reflect expert projections and professional consensus rather than empirical patient outcomes, prospective clinical trial data, or real-world intervention efficacy. High consensus does not guarantee clinical safety or diagnostic validity.
2. Expert Panel Selection and Convergence Bias: Although the panel achieved multidisciplinary representation, the initial sample pool was subject to self-selection bias among professionals with an existing interest in digital innovation. Furthermore, the Delphi technique inherently prioritizes consensus convergence, which risks marginalizing unconventional or dissenting clinical perspectives regarding the risks of immersive healthcare.
3. Baseline Technological Familiarity: The demographic protocol did not systematically assess panelists’ quantitative baseline hours of direct hands-on experience with mixed-reality headsets or generative AI interfaces. Variations in personal technological fluency may have influenced individual ratings of feasibility and timelines.
4. Absence of Patient and Service-User Perspectives: The panel was restricted to clinical, engineering, ethical, and policy experts. The absence of individuals with lived experience of psychiatric disorders, mental health service consumers, and patient advocacy representatives constitutes an important structural constraint that future participatory co-design research must rectify.
5. Socioeconomic and Global Disparities: While geographically distributed across five continents, the panel was predominantly anchored in high- and upper-middle-income healthcare systems. The feasibility ratings do not reflect the acute infrastructural constraints, unstable internet bandwidth, and hardware prohibitive costs characteristic of low-resource public health settings.

### 6.2. Conclusions

This three-round Delphi inquiry provides an empirical synthesis of multidisciplinary expert perspectives on integrating AI and Metaverse Realities into DMHS. Addressing RQ1 (Technological Architecture), expert consensus prioritizes AI-enhanced intake screening and immersive VR assessments as the most technically feasible and clinically impactful modalities, while designating autonomous crisis interventions as an unacceptably high-risk application requiring permanent human oversight. Regarding RQ2 (Bioethical Governance), findings demonstrate that existing static compliance models are inadequate for immersive environments; instead, health systems require dynamic consent protocols, cryptographically secured biometric boundaries, and transparent algorithmic audits. In response to RQ3 (Implementation Barriers), the study identifies technological complexity and hardware disparities as potential drivers of widened healthcare inequities, necessitating subsidized infrastructure and culturally adapted software interfaces. To realize the benefits of AI and MR without compromising patient safety, health systems must adopt a DC strategy, systematically sensing algorithmic risks, seizing verified clinical applications, and reconfiguring institutional bioethics. Future research should prioritize prospective clinical trials, formal economic cost-effectiveness evaluations, and participatory co-design frameworks that incorporate patient voices into the development of immersive digital therapeutics.

## Figures and Tables

**Table 1:** Overview of key areas and discussions of qualitative analysis.

Key Areas	Description	Observations
Therapeutic Outcomes	Evaluates the potential for Gen-AI and the Metaverse to enhance the efficacy of therapeutic interventions by creating personalized, immersive environments.	The consensus among experts highlights the significant benefits of integrating these technologies to improve therapeutic outcomes, offering more tailored and engaging treatments. However, ethical considerations, particularly regarding data privacy and user consent, are paramount to ensure these technologies are implemented responsibly.
Accessibility and Inclusivity	Assesses how these technologies can make mental health support more accessible and inclusive by breaking down geographical and physical barriers to care.	The potential of AI and MR to democratize access to mental health services is vast. They can provide support to underserved populations and reduce the digital divide. Ensuring that interventions are culturally sensitive and accessible to diverse populations is crucial to equitably distribute the benefits.
Interdisciplinary Collaboration	Examines the need for collaborative efforts across various disciplines to address the complex challenges at the intersection of technology and mental health.	Collaboration across fields such as computer science, psychology, ethics, and healthcare is essential to foster innovation that is both technologically advanced and deeply attuned to human needs. This interdisciplinary approach can help in developing solutions that are practical, ethical, and effective.
Ethical Considerations	Focuses on addressing privacy, confidentiality, and user autonomy concerns in the integration of advanced technologies in mental health services.	Ethical frameworks must be developed and prioritized to address the challenges of privacy, informed consent, and the risk of exacerbating mental health disparities. These frameworks should be transparent and inclusive, ensuring that all stakeholders, particularly users, have a voice in shaping the future of mental health support.
Regulatory and Governance Models	Investigates the development of regulatory and governance models to support innovation while protecting users from potential harm.	Developing appropriate regulatory and governance structures is crucial for the safe implementation of these technologies. Mechanisms for user input and participation in governance processes are necessary to ensure that DMHS are guided by the needs and interests of users.
Longitudinal Research	Emphasizes the importance of ongoing research to understand the long-term impacts of Gen-AI and Metaverse on mental health outcomes.	Long-term studies are needed to examine both the therapeutic potential and the risks associated with these technologies. This research will inform ongoing development and ethical considerations, ensuring that the integration of these technologies continues to enhance mental health support while mitigating any adverse effects.
Implementation Challenges	Addresses the technical, infrastructural, and user acceptance challenges in deploying Gen-AI and Metaverse in mental health services.	Overcoming these challenges requires a collaborative approach involving diverse stakeholders from mental health services, technological development, and policy-making. Continuous research and dialogue are essential to effectively integrate these technologies into various clinical and therapeutic contexts, ensuring their practical and beneficial application.
Future Projections and Assumptions	Considering the future directions for research and development efforts, focusing on creating comprehensive ethical frameworks and ensuring equitable distribution of benefits.	Future research should prioritize adaptable ethical frameworks that address privacy, confidentiality, inclusivity, and user autonomy. Ensuring that the benefits of these technologies are equitably distributed requires addressing the digital divide and cultural sensitivity. Proactive efforts are needed to ensure these technologies enhance, rather than undermine, human well-being.

**Table 2:** Projections and future considerations, with impact and probability of happening.

ID	Projections	Impact	Probability
1	AI-Enhanced Initial Screening: AI algorithms will be utilized to analyze data from initial screenings, identifying subtle indicators of mental health issues that may be overlooked in traditional evaluations. These screenings will include analysis of voice patterns, facial expressions, and behavioral data collected through metaverse interactions.	4.1	74%
2	Comprehensive Assessment via VR: The metaverse will enable comprehensive assessments through immersive VR environments, allowing mental health professionals to observe and evaluate patients’ reactions to simulated scenarios that reflect real-life stressors and triggers.	3.9	81%
3	AI-Assisted Diagnosis: AI will assist in the diagnostic process by integrating data from comprehensive assessments, medical history, and psychosocial factors, and comparing them against standardized criteria like the Diagnostic and Statistical Manual of Mental Disorders, Fifth Edition (DSM-5) and International Classification of Diseases, Tenth Revision (ICD-10) to provide diagnostic recommendations.	4.7	86%
4	Dynamic Individualized Treatment Plans: AI will create and continually update individualized treatment plans based on real-time data and feedback from patients’ interactions within the metaverse. These plans will be adaptive, responding to changes in the patient’s condition and progress.	4.6	79%
5	Enhanced Multidisciplinary Collaboration: The metaverse will facilitate seamless collaboration among multidisciplinary healthcare teams through shared virtual workspaces, real-time data sharing, and AI-coordinated care plans.	4.7	75%
6	AI-Optimized Pharmacotherapy: AI systems will analyze patient data to optimize pharmacotherapy, predicting individual responses to medications and adjusting dosages in real time to maximize efficacy and minimize side effects.	4.1	84%
7	Virtual Reality Psychotherapy: Psychotherapy will be delivered through the metaverse, utilizing VR to create immersive therapeutic environments. This will include simulations for exposure therapy, virtual role-playing for social skills training, and guided meditation environments.	4.7	90%
8	AI-Guided Counseling and Support: AI-driven virtual counselors will provide continuous emotional support, guidance, and education to individuals and their families within the metaverse, accessible at any time.	4.1	64%
9	Virtual Emergency Services: AI and metaverse technologies will provide immediate virtual emergency services, where patients can interact with virtual first responders and mental health professionals in real time during a crisis.	4.3	87%
10	AI-Managed Crisis Hotlines and Support Lines: AI-powered chatbots and virtual agents will manage crisis hotlines and support lines, offering immediate, round-the-clock support and directing individuals to appropriate resources within the metaverse.	4.9	95%
11	Virtual Rehabilitation Programs: Rehabilitation programs will be conducted within the metaverse, offering virtual vocational training, social skills development, and independent living support through interactive and engaging platforms.	3.7	57%
12	AI-Facilitated Peer Support Networks: AI will help organize and manage virtual peer support groups within the metaverse, matching individuals with similar experiences and facilitating structured support sessions.	4.3	78%
13	Automated Relapse Prevention Strategies: AI systems will monitor patients’ activities and interactions within the metaverse, identifying early signs of potential relapse and providing proactive interventions and reminders.	3.7	60%
14	Longitudinal Impact Studies: Longitudinal research studies will be conducted within the metaverse to assess the long-term impacts of AI and immersive technologies on mental health outcomes, providing valuable data for ongoing development and ethical considerations.	3.6	74%

**Table 3:** Statistical overview of the collected discussion rounds, areas, and sub-areas.

Key Area	Sub-Area	Round 1 IQR	Round 1 Mean	Round 1 SD	Round 2 IQR	Round 2 Mean	Round 2 SD	Round 3 IQR	Round 3 Mean	Round 3 SD
Assessment and Diagnosis	AI-Enhanced Initial Screening	1	4.4	0.8	1	4.6	0.6	1	4.7	0.5
	Comprehensive Assessment via VR	1.3	3.9	1.1	1	4.4	0.9	1	4.5	0.8
	AI-Assisted Diagnosis	1.3	3.9	1	1	4.2	0.8	1	4.3	0.7
	Analysis of EHR, Patient History, Behavioral Data	1	4.3	0.9	1	4.5	0.7	1	4.6	0.6
	ML, DL, NLP, and CV Integration	1	4	1	1	4.2	0.8	1	4.3	0.7
Treatment Planning and Coordination	Dynamic Individualized Treatment Plans	1	4.1	0.7	0	3.9	0.7	0	4	0.6
	Enhanced Multidisciplinary Collaboration	1	4.2	0.8	0	4.1	0.7	0	4.3	0.6
	AI-Optimized Pharmacotherapy	1	3.6	1	1	3.7	0.9	1	3.8	0.8
Intervention and Therapy	Virtual Reality Psychotherapy	1	3.5	0.9	1	3.7	0.9	1	3.8	0.8
	AI-Guided Counseling and Support	1	3.9	0.9	2	3.9	0.9	1	4	0.8
	Virtual Therapists and AI-Guided Therapy Sessions	1	4	0.8	1	4.2	0.7	1	4.3	0.6
	Teletherapy and Digital CBT	1	3.9	0.9	1	4.1	0.8	1	4.2	0.7
Crisis Intervention	Virtual Emergency Services	2	3.1	1.1	1	3.4	1	1	3.5	0.9
	AI-Managed Crisis Hotlines and Support Lines	1	3.7	1	1	4.1	1	1	4.2	0.9
Rehabilitation and Recovery	Virtual Rehabilitation Programs	1.5	3.8	0.8	1.5	3.9	1	1.5	4	0.9
	AI-Facilitated Peer Support Networks	1	3.7	1	1	4.2	0.8	1	4.3	0.7
	Automated Relapse Prevention Strategies	1	3.9	0.9	1	4.2	1	1	4.3	0.9
	Longitudinal Impact Studies	1	4	0.9	1	3.5	1	1	3.6	0.9
Ethical and Privacy Concerns	Ensuring User Safety and Privacy	1	4.1	0.8	1	4.3	0.7	1	4.4	0.6
	Addressing Ethical and Legal Concerns	1	4	0.9	1	4.2	0.8	1	4.3	0.7
	User Autonomy and Informed Consent	1	3.9	0.9	1	4.1	0.8	1	4.2	0.7
Accessibility and Inclusivity	Bridging the Digital Divide	1	3.8	0.9	1	4	0.8	1	4.1	0.7
	Ensuring Cultural Sensitivity	1	3.7	1	1	4	0.9	1	4.1	0.8
	Equitable Distribution of Benefits	1	3.9	0.9	1	4.1	0.8	1	4.2	0.7

## Data Availability

The raw qualitative transcripts and individual expert rating datasets generated and analyzed during the current study are not publicly available due to ethical and privacy restrictions, as well as participant confidentiality agreements governing expert panel feedback. De-identified, aggregated minimal datasets and consensus evaluation matrices supporting the findings of this study are available from the corresponding author upon reasonable academic request.

## Supplementary Materials

The following supporting information can be downloaded at: https://www.mdpi.com/article/10.3390/healthcare14203405/s1, File S1: Delphi Round 1 Qualitative Protocol and Expert Guide; File S2: Visual Scenario Construction, Prompt Engineering Registry, and Stimulus Neutrality Protocol.

## Appendix A

**Participant Information Sheet and Informed Consent Form**

**Study Title**: Exploring the Intersection of Artificial Intelligence and Metaverse Realities: Implications for Digital Mental Health Services

**Lead Investigator**: Antonio Pesqueira, PhD

**Affiliation**: ISCTE-IUL University of Lisbon

**Contact Email**: antonio_pesqueira@iscte-iul.pt

1. **Purpose of the Study**
  You are invited to participate in a research study examining the clinical, ethical, and practical implications of integrating Artificial Intelligence (AI) and Metaverse Realities (MR) into Digital Mental Health Services (DMHS). The objective of this study is to synthesize expert consensus on technological feasibility, governance frameworks, accessibility, and therapeutic utility to inform future clinical frameworks and policy guidelines.
2. **Study Procedures**
  Participation entails engaging in an iterative three-round Delphi inquiry:
  - **Round 1**: Responding to open-ended qualitative prompts regarding opportunities, ethical risks, and implementation barriers in AI- and MR-driven mental health support.
  - **Round 2**: Evaluating and rating structured statements, projections, and standardized clinical visual scenarios using Likert scales.
  - **Round 3**: Reviewing aggregate panel feedback to rank priorities, re-evaluate items, and solidify consensus across key DMHS domains.
  Each round is estimated to require approximately 20–30 min to complete.
3. **Voluntary Participation and Dynamic Consent**
  Your participation is entirely voluntary. Under our **Dynamic Consent** protocol, you will be re-notified before each sequential Delphi round, providing you with continuous autonomy to review, modify, or reaffirm your consent preferences as the study evolves. You may choose to skip any questions or withdraw from the study at any point without providing a reason and without any penalty or detriment to your professional standing.
4. **Data Confidentiality, Pseudonymization, and Security**
  - **Pseudonymization**: All qualitative insights, quantitative scores, and demographic information will be pseudonymized and assigned a unique alphanumeric code. Any direct identifying details will be permanently detached from the analysis.
  - **Data Storage**: All research data, transcripts, and records will be stored in secure, password-protected repositories using end-to-end encryption protocols. Access is strictly limited to the authorized research team.
  - **Reporting**: Results will be published exclusively in aggregate format or using de-identified excerpts, ensuring individual participants cannot be identified in publications or conference presentations.
5. **Potential Risks and Benefits**
  - **Risks**: There are no foreseeable physical or psychological risks associated with this study beyond the time invested in reviewing statements and visual scenarios.
  - **Benefits**: While there is no direct personal financial compensation, your participation contributes directly to shaping international research directions, clinical safety guidelines, and ethical standards for future digital mental health ecosystems.

**Informed Consent Declaration**

Please read each statement below and confirm your agreement:

- [ ] I confirm that I have read and understood the study information provided above.
- [ ] I have had the opportunity to consider the information, ask questions, and have received satisfactory responses.
- [ ] I understand that my participation is voluntary and that I am free to withdraw or modify my consent preferences at any time without consequence.
- [ ] I agree that my anonymized responses and ratings may be used in academic publications, reports, and presentations.
- [ ] I understand that all raw data will be stored securely using strict encryption and pseudonymization protocols.
- [ ] I freely consent to participate in this Delphi consensus research study.

**Participant Name (Printed):** _________________________________________________

**Participant Signature**: ______________________________________________________

**Date (DD/MM/YYYY)**: ________________________

**Researcher Name (Printed)**: _________________________________________________

**Researcher Signature**: _____________________________________________________

**Date (DD/MM/YYYY)**: ________________________
