# Supplementary material for: Exploring the Intersection of Artificial Intelligence and Metaverse Realities: Implications for Digital Mental Health Services
Source: Healthcare (Basel). 2026 Oct 12;14(20):3405. doi: 10.3390/healthcare14203405 (PMC13704517; doi:10.3390/healthcare14203405)
Supplement: Supplementary file 1 [file healthcare-14-03405-s001.zip › healthcare-4593972-supplementary.pdf]

Supplementary File S1

Delphi Round 1 Qualitative Protocol and Expert Guide

Study Overview and Preamble

Title: Exploring the Intersection of Artificial Intelligence and Metaverse Realities: Implications for Digital Mental Health Services (DMHS)

Target Panel: Multidisciplinary experts across Clinical Psychiatry, AI/MR Software Engineering, Digital Bioethics, and Healthcare Policy.

Protocol Description:

Round 1 of this Delphi inquiry utilizes an open-ended, semi-structured qualitative questionnaire designed to elicit unconstrained expert perspectives on the technical feasibility, bioethical requirements, clinical risks, and governance frameworks associated with integrating Artificial Intelligence (AI) and Metaverse Realities (MR) into mental healthcare. Responses gathered in this phase undergo rigorous reflexive thematic coding (Braun & Clarke framework) to establish the clinical sub-areas and scenario parameters evaluated in subsequent Delphi rounds.

Estimated Completion Time: 35–45 minutes.

Administration Mode: Asynchronous, secure web-based qualitative capture platform.

Participant Profile and Baseline Demographics

1. Primary Professional Discipline:

[ ] Clinical Psychiatry / Psychotherapy

[ ] AI / Machine Learning Software Engineering

[ ] Metaverse / Mixed Reality / Spatial Computing Development

[ ] Digital Health Bioethics / Healthcare Law

[ ] Healthcare Policy / Clinical Administration

2. Years of Active Professional Experience: ______ years

3. Primary Institutional Affiliation and Country of Practice: _________________________

4. Self-Assessed Hands-on Experience with Immersive Virtual Reality (XR) Platforms:

[ ] None / Minimal (<10 hours total)

[ ] Moderate (10–50 hours, occasional user)

[ ] Advanced (>50 hours, regular clinical, development, or research use)

5. Self-Assessed Familiarity with Generative AI / Large Language Model Tools:

[ ] Basic conceptual knowledge

[ ] Practical user experience (prompting, direct workflow usage)

[ ] Technical/Algorithmic expertise (model development, deployment, fine-tuning)

3. Semi-Structured Qualitative Inquiries across Core Conceptual Domains

Domain A: Clinical Affordances, Workflows, and Diagnostic Utility

Item A1: In your expert view, which specific mental health workflows (e.g., initial intake screening, differential diagnosis, active psychotherapeutic intervention, relapse prevention) offer the most compelling clinical rationale for the integration of AI analytics and immersive virtual reality environments? Please specify why.

Item A2: How do you perceive the diagnostic reliability of behavioral and affective data (such as vocal biomarkers, facial micro-expressions, avatar gaze tracking, and virtual proxemics) captured within immersive metaverse spaces compared to traditional face-to-face clinical assessments?

Item A3: What specific psychotherapeutic modalities (e.g., cognitive behavioral therapy, graduated exposure therapy, dialectical behavior therapy, social skills training) are best suited for delivery within mixed-reality metaverse settings, and what are the primary clinical contraindications?

Domain B: Autonomous Systems, Algorithmic Triage, and Clinical Safety

Item B1: Proponents suggest that generative AI conversational agents could manage acute crisis support hotlines or emergency psychiatric triage. What are the most significant clinical, diagnostic, and patient-safety hazards associated with deploying AI agents in high-risk psychiatric crises?

Item B2: What architectural safeguards or 'human-in-the-loop' mechanisms must be mandated before an AI system is permitted to analyze patient distress markers or suggest modifications to psychiatric treatment plans?

Item B3: In the event of an adverse clinical event (e.g., algorithmic hallucination delivering harmful advice, failure to detect acute suicidality during an immersive session), where does clinical, legal, and operational accountability reside?

Domain C: Bioethical Governance, Digital Phenotyping, and Dynamic Consent

Item C1: Immersive metaverse environments can continuously harvest real-time biometric and physiological data (termed 'digital phenotyping'). What specific bioethical frameworks are necessary to safeguard patient autonomy and prevent exploitative profiling in mental health applications?

Item C2: Traditional static, one-time informed consent models are widely viewed as inadequate for dynamic, AI-driven virtual environments. How should a 'dynamic consent' model be structured to allow patients real-time control over their behavioral data, session logs, and avatar telemetry?

Item C3: What specific architectural standards (e.g., local edge-processing, zero-knowledge proofs, end-to-end encryption) should be legally mandated to guarantee that highly sensitive psychiatric data is protected from commercial exploitation or unauthorized surveillance?

Domain D: Socio-Technical Disparities, Accessibility, and Organizational Agility

Item D1: How might high-cost virtual reality hardware, requirements for high-speed fiber-optic bandwidth, and technological literacy barriers impact equitable access to digital mental health services across socioeconomically disadvantaged or rural populations?

Item D2: Drawing upon the concept of organizational dynamic capabilities (the ability of a healthcare system to systematically sense innovations, seize clinical opportunities, and reconfigure institutional practices), what structural changes are required for hospitals and outpatient clinics to integrate AI and mixed reality safely into routine care?

Item D3: What closing recommendations or unaddressed concerns do you consider paramount for international regulatory bodies (e.g., FDA, European Medicines Agency) evaluating AI-driven spatial computing software as a medical device (SaMD)?

Supplementary File S2

Visual Scenario Construction, Prompt Engineering Registry, and Stimulus Neutrality Protocol

Methodological Rationale and Prompt Engineering Architecture

Six visual scenarios were rendered using the generative diffusion engine Midjourney v5.2 (Midjourney Inc., San Francisco, CA, USA). Following methodological standards for prompt engineering in psychological research (Dahò & Caci, 2026), prompts were constructed using a systematic, reproducible formula designed to prevent emotional, valence, or aesthetic framing bias:

Prompt Formula:

prompt: [Clinical Context & Architecture] + [Actor / Patient Interaction] + [Technological / Modality Specification] + [Aesthetic & Lighting Constraints] + [Fixed Engine Parameters]

Engine Configuration Parameters Held Constant Across All Stimuli:

Aspect Ratio: `--ar 16:9` (Standardized widescreen clinical display format)

Stylize: `--s 50` (Low stylization parameter to suppress artistic fantasy interpretation and maximize realistic photographic accuracy)

Chaos: `--c 0` (Zero chaos to ensure prompt determinism, high coherence, and reproducibility)

Quality: `--q 1` (Standard high-resolution diffusion pass)

Engine Version: `--v 5.2` (Fixed algorithm release)

Complete Registry of Clinical Scenarios and Prompt Syntax

Scenario 1: AI-Assisted Psychiatric Intake and Screening

Clinical Objective: Contextualize initial diagnostic data gathering, speech biomarker tracking, and automated symptom screening via an unobtrusive conversational interface.

Exact Prompt Syntax:

prompt: A clinical psychology consultation room, modern minimalist Scandinavian design, neutral beige and soft gray walls, large window with diffused overcast natural daylight. A female clinical psychologist in professional attire seated opposite an adult patient, engaged in calm dialogue. A subtle, non-intrusive semi-transparent holographic interface floats beside the clinician displaying neutral data metrics and acoustic waveforms. Photorealistic, documentary style, matte finish, balanced composition, no dramatic shadows, emotionally neutral atmosphere --ar 16:9 --s 50 --c 0 --v 5.2`

Midjourney Seed Number: `2849104821`

Scenario 2: Immersive Virtual Reality Exposure Therapy (VRET)

Clinical Objective: Contextualize a controlled, clinician-guided virtual exposure session for anxiety and trauma recovery.

Prompt Syntax:

/imagine prompt: A specialized outpatient clinical therapy suite with muted warm lighting, neutral wooden flooring, and soundproof wall panels. A male patient seated comfortably in an ergonomic armchair wearing a lightweight commercial mixed reality headset. Beside him, an experienced female clinical therapist holds an operational tablet monitor, calmly supervising the session. Clean clinical setting, natural lighting, documentary photography, no high-contrast drama, realistic proportions, calm medical atmosphere --ar 16:9 --s 50 --c 0 --v 5.2`

Midjourney Seed Number: `1940285719`

Scenario 3: Multidisciplinary Virtual Case Conference in the Metaverse

Clinical Objective: Contextualize cross-institutional clinical collaboration, remote diagnostic review, and AI-synthesized EHR visualization among healthcare providers.

Exact Prompt Syntax:

imagine prompt: A professional virtual collaborative medical conference room. Four photorealistic professional avatars representing a psychiatrist, a clinical neuroscientist, an ethicist, and a psychiatric nurse seated around an oval conference table. In the center of the table floats a neutral, three-dimensional spatial data visualization of a neurological diagram and anonymized health records. Balanced cool white office illumination, clear corporate-clinical aesthetics, functional workspace, highly professional, neutral valence --ar 16:9 --s 50 --c 0 --v 5.2

Midjourney Seed Number: `3857102944`

Scenario 4: AI-Guided Cognitive Behavioral Therapy (CBT) Micro-Intervention

Clinical Objective: Contextualize an outpatient user engaging with an adaptive generative AI conversational agent for structured cognitive reframing exercises.

Exact Prompt Syntax:

prompt: A domestic living room study area during daylight hours, organized and uncluttered, neutral muted tones. An individual seated at a desk calmly interacting with a desktop spatial computing display. On the display, an empathetic, dignified, photorealistic virtual avatar clinician delivers structured psychoeducational content. Realistic lighting, candid architectural photograph, soft shadows, neutral emotional expression, balanced tones --ar 16:9 --s 50 --c 0 --v 5.2`

Midjourney Seed Number: `4192840182`

Scenario 5: Immersive Group Therapy and Peer Support Space

Clinical Objective: Contextualize peer connection, communal scaffolding, and moderated group psychotherapy in an expansive, calming spatial reality.

Exact Prompt Syntax:

imagine prompt: An expansive, serene virtual therapeutic pavilion with biophilic architectural design, minimalist limestone columns, and a panoramic background of calm water and distant green hills under a soft overcast sky. Six diverse adult avatars seated in a relaxed circle on simple neutral seating, engaged in a group discussion led by a therapist avatar. Soft ambient daylight, gentle natural textures, photorealistic architectural rendering, peaceful and balanced, zero surrealism, non-distracting --ar 16:9 --s 50 --c 0 --v 5.2`

Midjourney Seed Number: `1048572911`

Scenario 6: AI-Managed Crisis Support and Triage Dashboard

Clinical Objective: Contextualize high-risk acute psychiatric crisis detection, real-time natural language sentiment flagging, and escalation protocols to human clinicians.

Exact Prompt Syntax:

imagine prompt: An emergency psychiatric crisis monitoring center workstation. A licensed mental health professional wearing a headset monitors a dual-screen digital dashboard displaying real-time text transcripts, automated linguistic risk stratifications, and triage priority queues. The interface uses clear clinical typography, standard medical green, amber, and blue status markers without flashing alarms or sensational colors. Clean office environment, realistic fluorescent lighting, focused professional setting --ar 16:9 --s 50 --c 0 --v 5.2`

Midjourney Seed Number: `2749103955`

3. Independent Pre-Evaluation Protocol for Stimulus Neutrality

To eliminate affective framing bias prior to panel distribution, all rendered scenarios were evaluated by an independent review committee of three senior academic clinicians (two board-certified psychiatrists, one senior clinical neuropsychologist). Reviewers were masked to the study's specific hypotheses and evaluated images across three validated visual psychophysical dimensions using 5-point semantic differential scales:

1. Affective Valence:

   Score 1: Highly Distressing / Negative / Threatening

   Score 2: Mildly Unpleasant / Somber

   Score 3: Strictly Neutral / Emotionally Equivocal

   Score 4: Mildly Uplifting / Pleasant

Score 5: Highly Euphoric / Excessively Optimistic

Target Tolerance Window: Mean score must fall within 3.0 ± 0.5 (2.50 to 3.50).

2. Visual Complexity:

Score 1: Sparse / Understimulating / Barren

Score 2: Minimally Detailed

Score 3: Balanced Architectural and Clinical Realism

Score 4: Moderately Cluttered

Score 5: Highly Chaotic / Overstimulating / Disorienting

Target Tolerance Window: Mean score must fall within 3.0 ± 0.5.

3. Clinical Plausibility / Ecological Realism:

Score 1: Science Fiction / Clinically Implausible

Score 2: Highly Unrealistic Clinical Workflow

Score 3: Moderately Plausible / Near-Term Feasible

Score 4: Highly Realistic Clinical Setting

Score 5: Indistinguishable from Actual Practice

Target Threshold: Mean score must be ≥ 3.50.

Pre-Evaluation Neutrality Results across Reviewers (R1, R2, R3):

Scenario ID	Valence (R1, R2, R3)	Valence Mean (SD)	Complexity Mean (SD)	Realism Mean (SD)	Neutrality Status
Scenario 1 (AI Intake)	3, 3, 3	3.00 (0.00)	2.67 (0.47)	4.33 (0.47)	Validated
Scenario 2 (VR Exposure)	3, 3, 4	3.33 (0.47)	3.00 (0.00)	4.67 (0.47)	Validated
Scenario 3 (Virtual Multidisciplinary)	3, 3, 3	3.00 (0.00)	3.33 (0.47)	4.00 (0.00)	Validated
Scenario 4 (CBT Agent)	3, 4, 3	3.33 (0.47)	2.67 (0.47)	3.67 (0.47)	Validated
Scenario 5 (Group Therapy)	4, 4, 3	3.67 (0.47)*	3.00 (0.00)	4.00 (0.82)	Prompt Adjusted
Scenario 5 (Regeneration pass 2)	3, 3, 3	3.00 (0.00)	3.00 (0.00)	4.00 (0.00)	Validated
Scenario 6 (Crisis Dashboard)	2, 3, 3	2.67 (0.47)	3.33 (0.47)	4.67 (0.47)	Validated

Note on Adjustment: Initial rendering of Scenario 5 scored slightly above the positive valence threshold (mean = 3.67) due to sunny landscape lighting. The prompt was revised to "soft overcast sky and diffuse ambient daylight," bringing the second iteration to strict neutrality (mean = 3.00). Inter-rater agreement across all final scenario ratings reached a Kendall's concordance coefficient of $W = 0.88$ ($p < 0.01$), confirming that the stimuli served as neutral visual prompts without introducing affective bias.
